# Impacts of COVID-19 on rural livelihoods in Bangladesh: Evidence using panel data

**DOI:** 10.1371/journal.pone.0259264

**Published:** 2021-11-29

**Authors:** Marcel Gatto, Abu Hayat Md Saiful Islam

**Affiliations:** 1 International Potato Center, Hanoi, Vietnam; 2 Department of Agricultural Economics, Bangladesh Agricultural University, Mymensingh, Bangladesh; Neijiang Normal University, CHINA

## Abstract

Rapid assessments have been emerging on the effects of COVID-19, yet rigorous analyses remain scant. Here, rigorous evidence of the impacts of COVID-19 on several livelihood outcomes are presented, with a particular focus on heterogenous effects of COVID-19. We use a household-level panel dataset consisting of 880 data points collected in rural Bangladesh in 2018 and 2020, and employ difference-in-differences with fixed effects regression techniques. Results suggest that COVID-19 had significant and heterogenous effects on livelihood outcomes. Agricultural production and share of production sold were reduced, especially for rice crops. Further, diet diversity and education expenditure were reduced for the total sample. Households primarily affected by (fear of) sickness had a significantly lower agricultural production, share of crop market sales, and lower health and education expenditure, compared to households affected by other COVID-19 effects, such as travel restrictions. In turn, (fear of) sickness and the correlated reduced incidence of leaving the house, resulted in higher off-farm incomes suggesting that households engage in less physically demanding and localized work. Policy-makers need to be cognizant of these heterogenous COVID-19 effects and formulate policies that are targeted at those households that are most vulnerable (e.g., unable/willing to leave the house due to (fear of) sickness).

## Introduction

COVID-19 has had devastating impacts globally. In economic terms, in 2020 the global economy contracted by 4.3% [1] and global employment reduced by an estimated 255 million full-time jobs [2]. Especially developing countries have been hit hard by the pandemic plunging an additional 88 to 115 million people into extreme poverty [3]. Declines in employment and income, jointly with only partially effective government support programs, and fall of living standards caused widespread food insecurity [4] and losses of income [5].

In curtailing the impact of the pandemic, national governments imposed several forms of movement restrictions which disrupted domestic and global agricultural value chains [6, 7]. A growing body of literature has begun to emerge on the impacts of COVID-19, the associated movement restrictions, and value chain disruptions, on reduced agricultural production and food and nutrition security for regions, such as Sub-Saharan Africa [8], Caribbean [9], Pacific Islands [10, 11], or individual countries, such as Bangladesh [12], China [13], Kenya [14], Peru [15], Myanmar [16, 17], or Nepal [18], Nigeria [19]. As study objectives were mainly to provide rapid assessments and viewpoints, evidence based on rigorous analyses on livelihood outcomes and household coping mechanisms are scant.

Overwhelming evidence exists regarding the negative effects on agricultural production and food and nutrition outcomes, other livelihood impacts are more complex and heterogenous in nature [20]. Take the example of labor effects. Urban workers who lost their employment and migrant workers from abroad returned home to their rural villages creating a labor surplus. At the same time, would-be migrant workers were restricted to travel to village communities creating labor shortages [21–23]. COVID-19 and movement restriction effects are gendered, too. Women were more severely affected in terms of job losses and the resulting losses in income compared to men, evidenced by a study from 6 countries. As a coping mechanism, women more often reduced food consumption but increased savings [24]. The pandemic created setbacks for women but has also created economic opportunities [25]. Another study from Bangladesh showed that women disproportionately more frequently left the house, mainly for grocery shopping, than men, despite movement restrictions [26]. Women may thus be more exposed to the risk of infection. In turn, this casts doubt on the effectiveness of lockdown regulations. The existing literature is limited in terms of evidence regarding the heterogenous effects of COVID-19 and the associated movement restrictions.

The objective of this paper is to provide rigorous evidence of the heterogenous effects of COVID-19 on several livelihoods outcomes for households living in rural Bangladesh. Particularly, we analyze how COVID-19 impacts household-level outcomes, such as agricultural production, gender-differentiated labor allocation, market sales, household expenditures, off-farm incomes, and food consumption. In doing so, we are particularly interested in examining the heterogenous effects of COVID-19, that is the effects of (fear of) sickness as opposed to other COVID-19 effects, such as travel restrictions and unemployment.

We use Bangladesh as a study case, in particular 2 districts in the southwest of the country. Bangladesh was in national lockdown from March-May 2020, which disrupted value chains and restricted movement of people and commodities [27]. This, in turn, negatively affected agricultural production and undermined food and nutrition security [26, 28, 29].

We use two datasets collected from 450 farming households from Satkhira and Khulna districts in 2018 and 2020. This allows us to compare outcomes from pre-COVID-19 (2018) with outcomes amid-COVID-19 (2020). Data for 2020 were collected in December after the lockdown. Methodologically, we utilize the panel nature of our data and use simple difference-in-difference analysis techniques with fixed effects. Therefore, unlike the existing evidence, our analysis provides nuanced evidence on the heterogeneous impact of COVID-19 and related measures on rural livelihoods in a developing country like Bangladesh.

## Background

On March 8, 2020, Bangladesh recorded the first person infected with COVID-19. Since then, infection rates climbed quickly which was accompanied by a high fatality rate. At the end of 2020, in Bangladesh a total of 510,000 people were infected by COVID-19 and 7,500 died [30]. The Khulna division, our study region, ranks third in terms of number of infections (25,000) only after Chattogram division (63,000) and Dhaka division (350,000) (idem). To slow the spread of the disease, the Bangladeshi government imposed a lockdown (March 24 –May 30) which drastically restricted movements of people and goods [27].

COVID-19 and associated lockdown had both severe direct and indirect effects on the population, penetrating several aspects of people’s livelihoods. In addition to the reported number of deaths, an increasing number of studies have been published on the effects of COVID-19 on mental health [31–33], especially for children [34], the garment industry and the resulting internal migration of more than 10 million of workers to rural areas [21, 35], as well as migration, reduced levels of remittances, and food insecurity for this vulnerable group [22, 36], and education [37].

The agricultural sector has also been severely affected by the impacts of COVID-19 resulting in disruptions in agricultural value chains and wide-spread food shortages. Disruptions, for example, left daily wage workers, who constitute one-third of Bangladesh’s total labor force, with reduced incomes and food insecure [38]. Labor shortages reduced agricultural production and movement restrictions limited access to markets for both sellers and buyers [29]. Prices for agricultural goods spiked at first but then quickly dropped sharply due to absence of buyers and traders in local markets, especially for perishable goods, such as vegetables and fish [39, 40]. In turn, in urban centers, prices for major food commodities drastically increased [12, 41]. The combination of reduced agricultural production and limited market access severely undermined food security and diet diversity [26, 28, 29].

Perceived compliance with government regulations were high (>90%) for adherence to social distancing, washing hands, and wearing of a mask [42]. However, impost movement restrictions were much less adhered to. Household members, mainly women, extensively left the house to purchase groceries for the family. This begs the question about the effectiveness of lockdown regulations, especially for movement restrictions [26].

**Fig 1:**
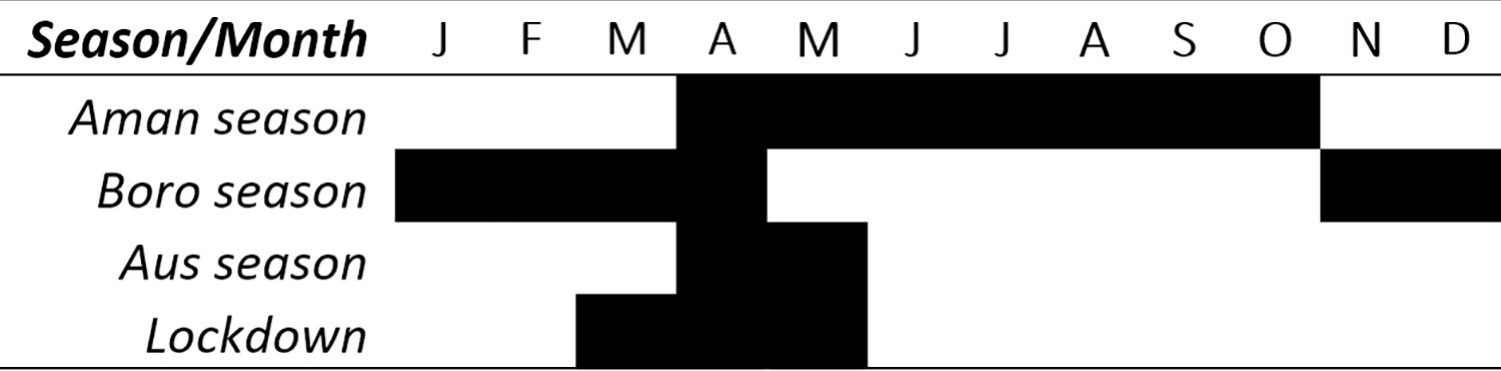
Agricultural seasons and lockdown 2020 in Bangladesh.

In Bangladesh, there are 3 main agricultural seasons, *aman* (May-Oct), *boro* (Nov-April), and *aus* (April-May). COVID-19 started in the end of March in 2020, and before the government-imposed lockdown and travel restrictions were in effect, much of the *boro* crop production in 2020 had already been harvested (Fig 1). For the *boro* crop harvest, such as *boro* rice or potato, the challenge was further downstream in the value chain. Traders were restricted to pick up the potatoes, farmers were limited to travel to markets to sell the produce. In turn, the *aman* crop planting, mainly rice, was severely affected by the government-imposed movement restrictions.

## Materials and methods

### Data

**Fig 2:**
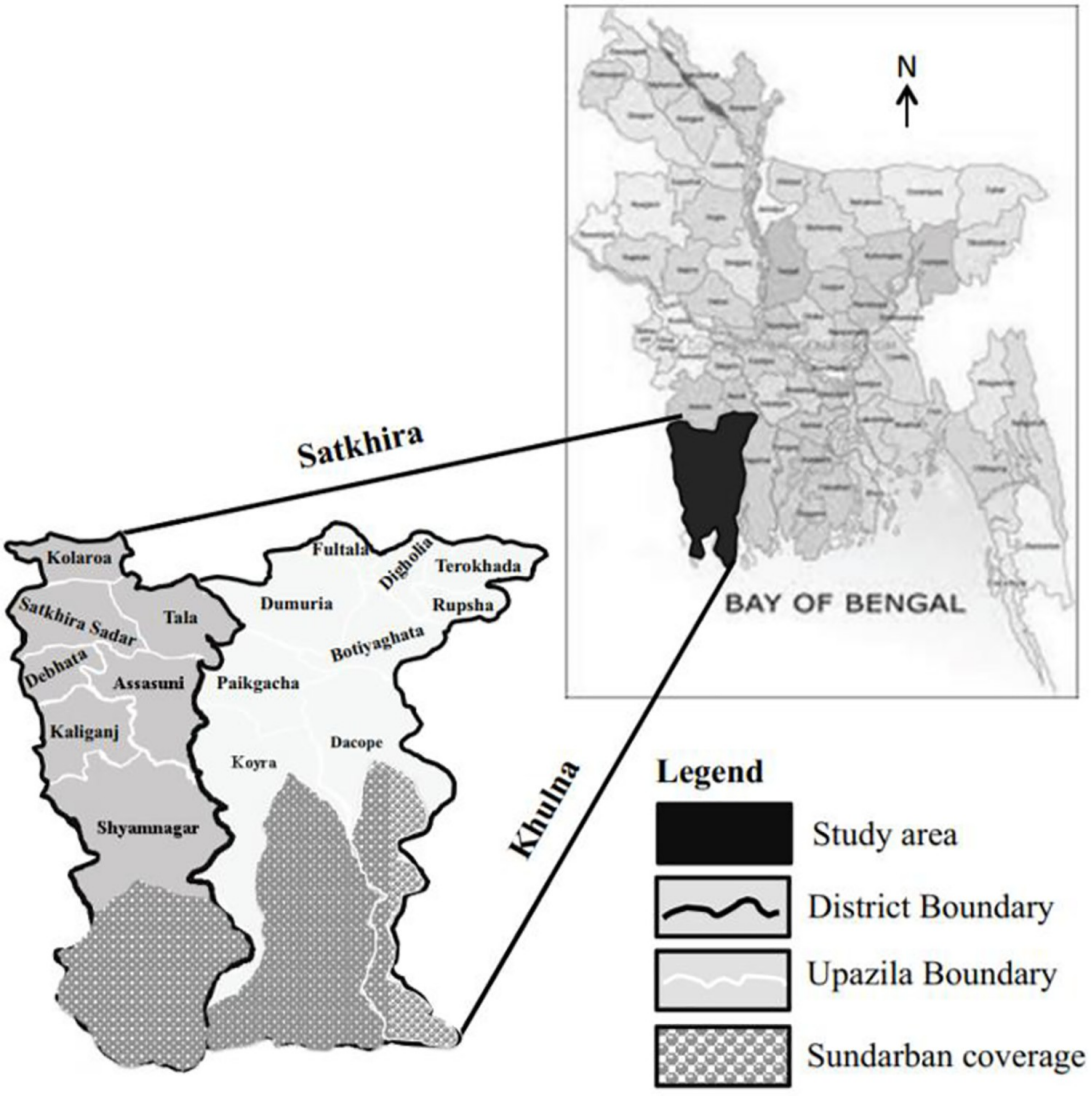
Study region in Bangladesh. Source: [43].

For this study, we use two rounds of data collected for the years 2018 and 2020. In December 2018, a first round of data collection was conducted in two districts–Satkhira and Khulna–in the Southwest of the country to establish a baseline (see Fig 2). These districts were part of a project to “Strengthen food systems resilience with salt tolerant potato and sweetpotato varieties” and increasingly affected by salinity intrusion. Six upazilas–the next lower administrative level–were purposively selected along a salinity gradient (i.e., high, medium, and low levels of salinity intrusion). To increase the likelihood of sampling potato- and sweetpotato-farming households, and households with pregnant/lactating women as well as infants (under 2 years of age), 9 unions–the next lower administrative level–were purposively selected using secondary data on the prevalence of those selection criteria. A total of 19 villages were randomly selected in those unions from complete village lists. Sampling proportional to size was used to randomly sample 450 households, oversampling households in villages with higher populations.

In December 2020, an endline survey was implemented, re-visiting households interviewed in 2018. Attrition was low, as only 10 households (2% of total sample) could not be identified. The total number of households interviewed in 2020 was thus 440. Taking together both rounds of data collection in 2018 and 2020, a balanced panel dataset was constructed with a total of 880 data points.

In both rounds, the same standardized questionnaire was used. In 2020, the questionnaire was amended with questions about the effects of COVID-19 in particular. Here we asked the respondents which aspect of COVID-19 had the highest impact on the respondent and the household. Possible answers were (1) unemployment/ loss of income, (2) shortages in food supply, (3) shops being closed, (4) travel restrictions, (5) social distancing, (6) quarantine or self-quarantine, (7) sickness or fear of getting sick, and (8) fear of dying. The questionnaires for both rounds are published along the corresponding datasets (see Data availability statement).

The survey was implemented using tablets, each interview lasted about 2 hours, and were generally conducted in the houses of the respondents. The main respondents were the head of the household who had overall knowledge about their families including agricultural production and marketing. However, consumption related questions were asked to female respondent who were in charge of the households’ meals. A total of 5 enumerators and two supervisors who were students at Bangladesh Agricultural University in Mymensingh, were identified and received intensive 1-week training during which the questionnaire was also piloted in the field and revised accordingly wherever necessary.

### Modelling effects of COVID-19

The objective of this research is to analyze the heterogenous impacts of COVID-19 on several household livelihood outcomes. We harness the panel structure of our data and specify the following fixed effects model to analyze the effects of the pandemic:

$$D_{i}=\alpha +\beta {Post}_{i}+\gamma {Sickness}_{i}+\delta {Post\;X\;Sickness}_{i}+\varepsilon_{i}$$ (1)

where *D* is one of the dependent outcome variables of interest of household *i*. For each dependent variable a separate equation is estimated. Outcome variables all represent key aspects of rural households, such as agricultural production and area, share of production sold at markets, labor allocation, expenditures, and consumption (i.e., diet diversity). *α* is the constant. The temporal variation is introduced by the dummy variable *Post_i_* which equals 1 for the ‘after COVID-19’ scenario and refers to the year 2020. The variable equals 0 for the year 2018 which represents the ‘before COVID-19’ scenario. We further use the variable *Sickness_i_* to introduce variation in the intensity of the COVID-19 effect. The variable equals 1 if COVID-19 caused an infection of household *i* or if the household was affected by (fear of) sickness. The variable equals 0 otherwise, which captures all other COVID-19 effects, such as travel restrictions, caused unemployment, etc. We predict that household member sickness is arguably the most severe case, as potentially all household members cannot work anymore and unable to travel to work, markets, etc. A coping strategy would be to employ more hired labor to perform essential on-farm tasks. There is thus a risk of crop losses potentially leading to reduced incomes from agricultural production with expected negative implications for household consumption and expenses. Depending on the course and severity of the infection, households could be affected for short or longer terms. While the effect of fear of sickness may not be as strong as sickness itself, we are unable to separate out those effects given how the question was asked. *ε_i_* is a random error term capturing the unobserved heterogeneity for household *i* that may affect the dependent variable(s). Households belonging to the same village were likely exposed to the same observed and unobserved heterogeneity. Thus, all regressions are estimated using robust standard errors that are clustered at the village level. The interaction term *Post X Sickness_i_* is the variable of interest as it captures the changes in livelihood outcomes due to ‘(fear of) sickness’. The interaction term in Eq (1) is a standard difference-in-differences estimator. As such, it takes the difference in outcomes observed in 2018 (‘before COVID-19’) and 2020 (‘after COVID-19-hit’) for households not affected by (fear of) sickness and subtracts it from outcomes observed for 2018 and 2020 for households affected by (fear of) sickness.

### Diversity index calculation

Consumption is proxied by the household diet diversity score (HDDS). This score measures the number of food groups–diversity–that a household consumed over a reference period (in our case during the past 24 hours). The HDDS is also often used as a proxy for household’s economic access to food and frequently used in food security and nutrition literatures [44, 45]. We established HDDS following the FAO guidelines [46] using 12 food groups, such as cereals, vegetables, oil and fats, potatoes, fish, cigarettes and other, eggs, sweets (sugar and sodas), legumes, fruits, milk and products, and meat.

### Ethical standard

High ethical standard was adhered to during the research. Official approval was not obtained through an Internal Review Board, however, this study was approved by the project and study teams of the International Potato Center. The study team maintained highest ethical standards using standardized questionnaire modules and informed consent forms which were used in other studies which have received official ethical clearance. Before each interview, the research objective, confidentiality, voluntary participation and anonymity of respondents were clearly explained. Verbal consent of each respondent was recorded.

## Results

### Descriptive statistics

#### Descriptive statistics for selected explanatory variables

The average household in our sample was 47.7 years old in 2018 and, thus, about 2 years older in 2020 (Table 1). Almost all household heads (98%) were male and married (96%). The number of household members were, on average, 4.42 in 2018 and about the same size in 2020. The total years of education were 5.65 in 2018 which was also about the same in 2020. About half of our sample (52%) was located in Khulna district as opposed to Satkhira district. At the time of the survey in 2020, about 26% of respondents reported to have fallen sick because of COVID-19 or feared getting sick. As mentioned earlier, unfortunately, it is not possible to separate out the effect of COVID-19-related sickness and the fear thereof. The remaining 74% of the sample reported to be primarily affected by travel restrictions and unemployment as a result of COVID-19.

**Table 1:** Descriptive statistics of selected explanatory variables.

	Before COVID-19 (2018)	After COVID-19 hit (2020)
	*Mean (Std*. *dev)*	*Mean (Std*. *dev)*
*Age*	47.7 (12.5)	49.2 (12.3)
*Male (dummy)*	0.98 (0.11)	0.97 (0.14)
*Married (dummy)*	0.96 (0.19)	0.96 (0.18)
*Household members*	4.42 (1.52)	4.57 (1.72)
*Years of education*	5.65 (4.39)	5.74 (4.36)
*Khulna district (dummy)*	0.52 (0.52)	0.52 (0.51)
*(Fear of) Sickness (dummy)*	0.00 (0.00)	0.26 (0.44)

#### Descriptive statistics for outcome variables

We now turn to the descriptive statistics of the dependent outcome variables which are summarized in Table 2. First, total agricultural production averaged 4.4t in 2018 before the COVID-19 pandemic. In 2020, after COVID-19 hit, agricultural production was reduced to 3.4t. Rice harvests for both *aman* and *boro* rice were significantly reduced by an average of 21% (0.31t) and 15% (0.25t), respectively, between 2018 and 2020. Other crops, such as potato, however, did not experience significant reductions. The observed reduction in agricultural production was likely associated with a reduction in area utilized for agricultural activities. While respondents utilized 172 decimal of land (or 0.71 ha) before COVID-19, land area was reduced by 37.1 decimal (21.5%) to 135 decimal (or 0.55 ha) in 2020.

**Table 2:** Descriptive statistics of outcome variables.

	Before COVID-19 (2018)	After COVID-19 hit (2020)	Difference test
	*Mean*	*Mean*	
*Agricultural production*			
Total harvest (t)	4.431	3.407	-1.03***
Aman rice harvest (t)	1.456	1.146	-0.31***
Boro rice harvest (t)	1.645	1.400	-0.25**
Potato harvest (t)	0.653	0.599	-0.05
Ag. area (decimal)	172.3	135.3	-37.1***
*Market sales (in %)*			
Aman rice sold	0.275	0.191	-0.08***
Boro rice sold	0.306	0.244	-0.06***
Potatoes sold	0.264	0.233	-0.03*
*Expenditure (in Tk)*			
Total exp.	132,649	150,776	18,127**
Food exp.	56,392	61,230	4,838***
Health exp.	8,279	12,894	4,615***
Education exp.	8,949	4,612	-4,337***
*Labor allocation (% of total labor)*			
Male hired	0.292	0.316	0.02*
Female hired	0.059	0.087	0.03***
Male family	0.509	0.410	-0.01***
Female family	0.139	0.142	0.003
*Food consumption*			
Diet Diversity (HDDS)	6.691	6.251	-0.44***
*Alternative income (in Tk)*			
Off-farm income	80,997	106,171	25,174***

Notes

***significant at the 1%-level

**significant at the 5%-level

*significant at the 10%-level. Tk = Bangladeshi Taka.

Second, before COVID-19, respondents sold 27.5% of their *aman* rice harvest and, correspondingly, kept 72.5% for own consumption. In 2020, this share decreased to 19.1%, a reduction of 8.4 percentage points. For *boro* rice, results are similar: in 2018, the share of *boro* rice harvest sold at markets was 30.6% which was reduced to 24.4 in 2020. Regarding potatoes, the share sold at markets was 26.4% which was only slightly reduced in 2020 to 23.3%, which corresponds to a reduction of some 3%.

Third, household expenditure increased significantly by 13.6% (from 132k to 150k Taka) between 2018 and 2020. In more detail, food and health expenditures increased significantly by some 9% and 13.5%, respectively, for the same period. Education expenditure, in contrast, dropped by some 48%, from 8.9k to 4.6k Taka, on average, between 2018 and 2020.

Fourth, in terms of labor allocation, households hired slightly more male (2.4%) and female (2.8%) labor, probably to compensate for the reduction in male family labor (9.9%). Female family labor, in contrast, did not decrease significantly between 2018 and 2020.

Fifth, in terms of food consumption, respondents did significantly reduce food diversity, as measured by the HDDS. Before COVID-19, households consumed food items from 6.69 categories while in 2020, the food diversity consumed was reduced to 6.25 categories, a slight decrease of 0.44 food categories.

Sixth, while agricultural production on-farm decreased between 2018 and 2020, households increased off-farm income by some 31% (from 81k to 106k Taka).

#### Descriptive statistics for household diet diversity

In terms of food consumption, we now turn to a detailed description of changes in consumption using the individual food item categories which, in turn, were used to create the HDDS (Table 3). Staple commodities, such as cereals (mainly rice) and vegetables were consumed by almost all respondents in both years. Next, an additional 4% of respondents increased consumption of oil and fats. Potatoes were the 4^th^ most important food item in 2018. A clear reduction in potato consumption by almost 12 percentage points was observed for the year 2020. In contrast, fish consumption increased by some 8 percentage points between 2018 and 2020. Fish production is commonplace in our study region and an increased consumption allows household members to stay at home rather than going to market to buy potatoes, for instance. Moreover, between 2018 and 2020 cigarette and consumption of sugars were reduced by 11% and 13%, respectively, likely because scarce funds were needed to purchase other more essential consumables. Consumption of other important and nutritious food item, such as legumes (-6.6%) and fruits (-9.0%), however, were also significantly reduced. The consumption of other food items which are important sources of micronutrients, such as meat, milk, and eggs, remained unchanged.

**Table 3:** Descriptive statistics of food categories used for Household Diet Diversity Score (HDDS).

	Before COVID-19 (2018)	After COVID-19 hit (2020)	Difference test
	*Mean*	*Mean*	
Cereals	100	100	0
Vegetables	97.1	97.2	0.1
Oil and fats	90.7	94.8	4.09***
Potatoes	85.1	73.4	-11.7***
Fish	74.2	82.1	7.89***
Cigarettes and other	60.7	49.5	-11.2***
Eggs	30.9	31.1	0.2
Sweets (sugar and sodas)	30.7	17.3	-13.4***
Legumes	30.2	23.6	-6.6**
Fruits	29.6	20.6	-9***
Milk and products	20.9	18.8	-2.1
Meat	19.1	16.3	-2.8

Notes

***significant at the 1%-level

**significant at the 5%-level.

### Regression results for heterogenous COVID-19 effects

We now turn to the regression results for heterogenous COVID-19 effects using household fixed effects. The interaction term *Post X Sickness* is the variable of interest. If the coefficient for this interaction term is negative and significant suggests that (fear of) sickness plays a more detrimental role than other effects of COVID-19, such as travel restrictions, caused unemployment, etc. The results are summarized in Tables 4–8.

**Table 4:** Regression results for COVID-19 effects on agricultural production outcomes.

	(1)	(2)	(3)	(4)	(5)
	Total harvest (kg)	Aman rice (kg)	Boro rice (kg)	Potato (kg)	Agricultural area (decimal)
*Post X Sickness*	-1314.8**	21.37	-267.6	-111.4	-28.89*
	(640.1)	(319.7)	(399.3)	(100.7)	(16.56)
*Sickness*	1192.1	-92.36	210.5	58.41	11.52
	(840.7)	(184.2)	(183.1)	(311.9)	(13.27)
*Post (year 2020)*	-693.1***	-314.7***	-178.3	-28.08	-30.39***
	(129.2)	(112.9)	(160.1)	(63.65)	(5.104)
*Constant*	4130.7***	1478.9***	1592.9***	639.4**	172.2***
	(440.2)	(251.1)	(194.8)	(260.4)	(17.92)
*R-squared*	0.018	0.008	0.004	0.001	0.015
*Household fixed effects*	YES	YES	YES	YES	YES
*Observations*	866	844	844	826	866

Notes: Robust standard errors clustered at the village level in parentheses

*significance at the 10%-level

**significance at the 5%-level

***significance at the 1%-level.

**Table 5:** Regression results for COVID-19 effects on labor allocation outcomes.

	(1)	(2)	(3)	(4)
	Female hired	Male hired	Female family	Male family
*Post X Sickness*	-0.027*	0.037	-0.041*	-0.022
	(0.019)	(0.031)	(0.022)	(0.025)
*Sickness*	0.004	-0.005	-0.003	0.004
	(0.011)	(0.022)	(0.013)	(0.018)
*Post (year 2020)*	0.035***	0.014	0.013	-0.094***
	(0.012)	(0.019)	(0.009)	(0.023)
*Constant*	0.058***	0.293***	0.139***	0.508***
	(0.008)	(0.022)	(0.012)	(0.021)
*R-squared*	0.019	0.004	0.007	0.04
*Household fixed effects*	YES	YES	YES	YES
*Observations*	866	866	866	866

Notes: Robust standard errors clustered at the village level in parentheses

*significance at the 10%-level

***significance at the 1%-level.

**Table 6:** Regression results for COVID-19 effects on market sales outcomes.

*Share of harvest sold at market (%)*	(1)	(2)	(3)
	Aman rice	Boro rice	Potatoes
*Post X Sickness*	-0.107*	-0.084**	-0.025
	(0.061)	(0.039)	(0.051)
*Sickness*	0.019	0.041	0.021
	(0.038)	(0.035)	(0.063)
*Post (year 2020)*	-0.058**	-0.041	-0.025
	(0.022)	(0.026)	(0.024)
*Constant*	0.271***	0.296***	0.258***
	(0.033)	(0.029)	(0.051)
*R-squared*	0.024	0.011	0.002
*Household fixed effects*	YES	YES	YES
*Observations*	844	844	826

Notes: Robust standard errors clustered at the village level in parentheses

*significance at the 10%-level

**significance at the 5%-level

***significance at the 1%-level.

**Table 7:** Regression results for COVID-19 effects on household expenditure (in log) outcomes.

	(1)	(2)	(3)	(4)
	Total	Food	Health	Education
*Post X Sickness*	-0.151	-0.709***	-0.644*	-0.671**
	(0.104)	(0.198)	(0.382)	(0.266)
*Sickness*	0.079	0.122**	0.125	0.391
	(0.049)	(0.053)	(0.121)	(0.372)
*Post (year 2020)*	0.141**	0.208***	0.341**	-0.542**
	(0.061)	(0.055)	(0.141)	(0.211)
*Constant*	11.57***	10.79***	8.219***	6.181***
	(0.054)	(0.051)	(0.086)	(0.287)
*R-squared*	0.008	0.057	0.011	0.009
*Household fixed effects*	YES	YES	YES	YES
*Observations*	880	880	880	880

Notes: Robust standard errors clustered at the village level in parentheses

*significance at the 10%-level

**significance at the 5%-level

***significance at the 1%-level.

**Table 8:** Regression results for COVID-19 effects on dietary diversity and off-farm income.

	(1)	(2)
	HDDS	Off-farm income (log)
*Post X Sickness*	0.206	1.132**
	(0.237)	(0.528)
*Sickness*	-0.007	-1.442**
	(0.128)	(0.589)
*Post (year 2020)*	-0.498***	0.171
	(0.175)	(0.316)
*Constant*	6.697***	8.219***
	(0.087)	(0.383)
*R-squared*	0.03	0.009
*Household fixed effects*	YES	YES
*Observations*	880	880

Notes: Robust standard errors clustered at the village level in parentheses; HDDS = Household Diet Diversity Score

**significance at the 5%-level

***significance at the 1%-level.

Agricultural production was significantly reduced because of COVID-19 and the effect is much stronger for households affected by (fear of) sickness, as the negative interaction term suggests. (Fear of) sickness reduced agricultural production by about 2t (-1,315 + (-0.693)) while households affected by other COVID-19 effects reduced agricultural production by 0.693t. However, the interaction terms enter insignificantly in the remaining estimations (2)-(4) depicted in Table 4. This suggest that for individual crops, changes in harvests were not significantly different for households primarily affected by (fear of) sickness and other COVID-19 effects.

Next, female hired labor was significantly lower for households which were affected by (fear of) sickness, as the significant and negative interaction term suggests (-0.027). This, in turn, means that the share of female hired labor increased by 0.8 percentage points (0.035 + (-0.027)) because of (fear of) sickness. In contrast, other COVID-19 effects resulted in an increase of female hired labour by 3.5 percentage points. Furthermore, the effect of (fear of) sickness was significantly lower in explaining female family labor, as the interaction term (-0.041) in estimation (3) of Table 5 suggests. Here, however, the effect of (fear of) sickness on female family labor is overall negative (0.013 + (-0.041) = -0.028), while other COVID-19 effects had a positive but insignificant effect (0.013). We further found no effect of COVID-19 on male hired labor allocation. In addition, male family labor was reduced by COVID-19 by some 9 percentage points. Here, the interaction term enters insignificantly, suggesting that the effect of (fear of) sickness was not different from other COVID-19 effects.

Regarding market sales, (fear of) sickness had overall stronger negative effects compared with other COVID-19 effects (Table 6), especially for rice. For instance, for *aman* rice, the interaction term is negative and significant (-0.107). Here, (fear of) sickness resulted in a reduction of rice sold at markets by 16.5 percentage points (-0.107 + (-0.058)), while other COVID-19 effects reduced the share of *aman* rice sold at markets by 5.8 percentage points. We found a similar effect for *boro* rice. Here, (fear of) sickness resulted in a reduction of *boro* rice sold at markets by 12.5 percentage points, while other COVID-19 effects were insignificant. Also, the results suggest that COVID-19 did not have a significant effect on potato sales but the coefficients have the expected negative sign.

Regarding household expenditure, we did not find a heterogenous COVID-19 effect on total expenditure, as the insignificant interaction term coefficient suggests (Table 7). Households affected by other (than (fear of) sickness) COVID-19 effects, however, increased household expenditure (0.141). For individual expenditure items we found heterogenous COVID-19 effects. In particular, food and health expenditure decreased significantly in 2020 for households affected by (fear of) sickness while expenditure increased for households affected by other COVID-19 effects. Education expenditure, in contrast, was reduced for all households, but to a larger extent for households affected by (fear of) sickness (-0.671 + (-0.542)).

In terms of food consumption, we did not find a heterogenous COVID-19 effect, as the insignificant interaction term coefficient suggests in estimation (1) of Table 8. However, as the coefficient for Year 2020 enters negatively, is indicative of an overall negative effect of COVID-19 on diet diversity. Households reduced diet diversity by some 0.5 food categories.

We also found heterogenous COVID-19 effects for off-farm income (Table 8). (Fear of) sickness increased off-farm income (1.132 + 0.171) while the effect of other COVID-19 effects on off-farm income is insignificant.

## Discussion

First, in terms of agricultural production, harvests were significantly reduced, as studies extensively suggests [19, 29]. Especially harvests for *aman* rice, Bangladesh’s staple crop, were substantially reduced. That we only found a negative effect for *aman* rice (and not for *boro* rice) may be explained by the timing of the pandemic and associated government restrictions. The lockdown measures of April 2020 affected harvesting and post-harvest activities for *boro* rice. For *aman* rice, in contrast, the planting period was affected possibly resulting in reduced labor availability. In addition, (fear of) sickness affected farmers’ willingness and ability to travel to their plots during planting stages. But the effect of (fear of) sickness was not more severe than other effects, such as travel restriction, as our results show. How lockdown and other government measures affect different stages of crop production would be an important avenue of future research. In addition, government agricultural support programs, which provided inputs, such as improved varieties [42] may have resulted in productivity gains to offset the negative effects associated with the lockdown. These support programs, however, were limited to specific areas and not implemented in our study area. The coefficient explaining potato harvests entered insignificantly which is likely due to small sample size. However, the coefficient has the expected negative sign. Related to the observed reduction in agricultural production is the finding that agricultural area utilized was reduced significantly. (Fear of) sickness resulted in a much more substantial reduction. These households utilized only some 66% ((29+30) / 172 decimal) of their agricultural land in 2020 while the other COVID-19 effects resulted in a utilization of 82% of agricultural land. Likewise, total harvests were also significantly lower for households that were primarily affected by (fear of) sickness. This was expected as infection and the fear thereof reduced the ability and willingness of households to perform agricultural work or travel to markets to sell produce.

We further analyzed the effects of COVID-19 on household expenditure. Here, we found an insignificant effect of (fear of) sickness while other COVID-19 effects increase expenditure. This is in line with the prediction that (fear of) sickness affects the ability and willingness to leave the house as frequent as, for instance, travel restrictions would. Leaving the house less frequently reduces market participation and thus overall expenses. Household members that left the house, however, were faced with higher food and health expenditure, as our results suggest. Those who did were affected by higher prices for key staples (see S1 Table). Price increases, however, are commodity and location dependent. For instance, the price for perishable goods such as vegetables, fish, and chicken declined sharply due to lack of buyers and traders in local markets, or rumors regarding food safety concerns [29, 39]; in urban areas, in turn, prices for major food commodities, such as rice and fish, have spiked [12, 41]. Household members who traveled were also likely to incur higher health expenditures because they visited doctors and pharmacies. In addition, these findings suggest that households affected by travel restrictions left their homes more often than households affected by (fear of) sickness, despite travel restrictions. This is in line with other research that found that mainly women left the house, particularly, to buy groceries for the family [26]. Unfortunately, we cannot confirm that mainly women left the house and were thus more exposed to the threat of infection than men. More gender-disaggregated data is needed to confirm this. Moreover, additional unexpected costs were, sometimes involuntarily, compensated for by a significant reduction in education expenditure. Note that it is not clear if the reduction in education expenditure was due to costs-savings caused by government-imposed school closures [47] or if children of school age had to (temporarily) drop out of school to support the household, as other research has found [48], or both. In either case, the observed reduction in education expenditure is indicative of lower rates of children attending schools. This is confirmed by other studies (e.g., [5]).

Another impact of COVID-19 relates to changes in market exchanges. In particular, households significantly reduced the amount of agricultural production that was sold at local markets. This was the case for *aman* rice. Probably, the uncertainty associated with the pandemic, its duration, and linked limited access to food, induced households to keep a higher share or production for own consumption. Nevertheless, the results also suggest that households continued to sell a substantial part of their produce, possibly driven by fear of losing income and higher prices for staple commodities, such as rice (see S1 Table) [41]. In doing so, household members were required to leave the house. (Fear of) sickness resulted in keeping a larger share of *aman* and *boro* rice for potential own consumption. Given the findings for *aman* rice and the significance at the 10-level (*P = 0*.*130*) found for the *boro* coefficient, we argue that COVID-19 effects, such as travel restrictions and caused unemployment had a similarly negative effect for share of *boro* rice sold at markets as (fear of) sickness. Next, the insignificant results for share of potato sold at markets may be indicative of potato being an important cash crop. It would be worth exploring if and how potatoes contribute to household resilience in Bangladesh, as it has been found for other roots and tuber crops in the Philippines during climatic shocks [49].

Further, COVID-19 also affected labor allocation. Irrespective of the severity of COVID-19 impact, male family labor was reduced substantially. One explanation could be that increased involvement of hired labor freed-up time resulting in a reduction of male family labor. Possibly, male household members fell sick more often. Another possibility could be that the freed-up labour was used for other off-farm income-generating activities which did also increase significantly between 2018 and 2020. (Fear of) sickness also reduced female family labor. The same reasons as above apply. Moreover, the involvement of male hired labor did not change which suggests that supply of male labor was not disrupted. In contrast, more female hired labor was utilized, with a lower involvement observed for households affected by (fear of) sickness. This finding is in line with the ‘internal migration’ hypothesis stating that due to substantive job losses in urban areas, mainly in the garment industry which mainly employs women, people returned to their villages which, in turn, had a positive effect on the availability of (mainly female) labor in rural areas. As a result, wages for day laborers may have experienced a drop, as research suggests for our study districts [38].

Off-farm income did only significantly increase for households affected by (fear of) sickness, the other households continued as before the pandemic. This finding suggests that travel restrictions were not fully adhered to and even (fear of) sickness was not an impediment. While off-farm income may stem from activities in and around the own village community, these could also stem from opportunities farther away. What remains puzzling is how, on the one hand, agricultural production and male family labor was reduced, suggesting that (fear of) sickness was a restrictive factor for leaving the house. While, on the other hand, off-farm income was increased which suggests that household members affected by (fear of) sickness did actually leave the house more frequently. We conjecture that this seeming contradiction may be a matter of timing. Possibly, the reduction of agricultural production and labor occurred in the beginning of the lockdown when *aman* rice was planted. After the lockdown was lifted and the fear of sickness normalized, households were possibly more able and willing to leave the house/travel to engage in off-farm activities. Households that we affected by other COVID-19 effects did not (need to) change in this respect.

A final studied effect was on changes in food consumption, in particular on diet diversity. The consumption of food from 6.25 categories observed for 2020 and the reduction by 0.45 categories is in line with other recent studies on food diversity and insecurity due to COVID-19 in Bangladesh [26, 28]. We further showed several consumption adjustments. On a positive note, households reduced consumption of cigarettes and other ‘luxury’ items and sugary products, such as sodas or sweets. But likewise, the consumption of nutritious food items, such as legumes, potatoes, and fruits were reduced. More households, in turn, used more oil and fats and fish. With the fish value chain also being severely affected by disruptions [29], fish was abundantly available, especially in our coastal study districts. A deeper analysis that examines if the increases in some consumption items were able to offset the nutritional content of items that were reduced, will be an important avenue of future research. We further found that diet diversity was reduced more for households affected by other (than (fear of) sickness) COVID-19 effects. This, again, seems puzzling as we predicted that being unable to leave the house will reduce household access to (diverse set of). On the other hand, the size of the effect should not be over-interpreted which was less than 0.5 categories.

## Conclusions

Various studies that mostly reviewed and aggregated findings predicted that COVID-19 and related measures will affect local food system in low and middle income countries [50]. In this study, we present rigorous evidence of the impacts of COVID-19 on several livelihood outcomes using panel data from pre-COVID-19 (2018) and after COVID-19 hit (2020). We found that COVID-19 and related government restrictions had a significant impact on rural livelihoods in Bangladesh and that (fear of) sickness had a more severe effect on livelihood outcomes than other COVID-19 effects. Likely, the (fear of) sickness reduced the ability and/or willingness of household members to leave the house.

Taken together, we found that (fear of) sickness had a more severe effect on food security, compared with other COVID-19 effects. Agricultural production of staple food items was significantly reduced which households partly compensated for by reducing the amounts of produced staples sold at markets. Lower ability/willingness to leave the house resulted in reduced expenditure on food items, in particular those that are generally purchased at markets (such as fruits). Moreover, (fear of) sickness and the associated reduced ability/willingness to leave the house reduced health expenditures possibly resulting in lowered health outcomes compared with households affected by other COVID-19 aspects, such as travel restrictions. For home-bound households, children also had to support the family more extensively who, in turn, did more often not attend school, as the reduction in education expenditure suggests. The same households were able to engage in more off-farm income generating activities than those households that were affected by other COVID-19 effects. These were likely activities that are less physically taxing and available in the community to avoid the need to travel. Despite households primarily affected by (fear of) sickness adopted strategies to mitigate the pandemic’s effects, we conclude that those households were overall more severely affected by COVID-19 than households that were affected by other effects, such as travel restrictions.

This study has limitations. Key to analyzing the heterogenous COVID-19 effects is to adequately distinguish between sickness, fear of sickness, and other COVID-19 related impacts. In our study, we used self-reported impacts of COVID-19. Naturally, respondents who must travel much for work, for instance, may be more likely to report that travel restrictions were an issue than other aspects of COVID-19, such as fear of sickness or shops closures. In turn, respondents that do not have to travel much for work, are more likely to report that other than travel restrictions were an issue, such as (fear of) sickness. Add to this that effects of COVID-19 were likely different for household members, in particular men and women, as evidence suggests [26]. More research is warranted that is able to exogenously attribute COVID-19 effects to sickness (or infection), fear of sickness, travel restrictions, etc. and, in addition, takes an intra-household gender-differentiated approach.

Awareness needs to be created among policy-makers that (fear of) sickness effects were more detrimental for livelihood outcomes, mainly food security, health, and education than other COVID-19 effects, such as travel restrictions. Policy-makers are thus advised to prioritize households infected by COVID-19 in their support programs.

Government support programs could be related to agricultural production and involve labor-saving farming practices and productivity enhancing technologies. The Bangladesh government, for instance, has rolled-out a mechanization support program to help farmers with the *aman* rice harvest [51]. In other areas, improved (*boro*) rice varieties were disseminated to increase yields [52]. These rapid government interventions are important but need to be scaled to other areas, crops, and target vulnerable households (i.e., infected by COVID-19). Other ‘infection safe’ policy interventions could aim at shortening the marketing channels within value chains [20]. For instance, producer organizations in Bangladesh have created online fish marketplaces where fish can directly be sold and bought from the nearest farm [12]. Buyers and sellers do not need to travel to crowded wet markets which reduces the risk of infection drastically. In a similar vein, further localizing food production and exchange of food by introducing community marketing schemes could effective [53]. Another effective intervention to reduce the need to travel to markets is the use of improved storage which has been found to contribute to food security [54].

Every crisis produces winners and losers. Producing rigorous evidence of the impact of global threats, such as a pandemic, is key to support or refute anecdotal evidence, much of which was produced especially in the beginning of the crisis. In addition, acknowledging that specific groups of people, such as those that have already been infected by COVID-19 or have relative more fear of being infected (possibly due to existing comorbidities) behave in a way that results in higher vulnerabilities, is crucial for designing and targeting effective interventions including social protection programs.

## Supporting information

S1 Table Prices for selected key agricultural goods in 2018 and 2020. (DOCX)
