# Supplementary material for: Impacts of COVID-19 on rural livelihoods in Bangladesh: Evidence using panel data
Source: PLoS One. 2021 Nov 29;16(11):e0259264. doi: 10.1371/journal.pone.0259264 (PMC8629178; doi:10.1371/journal.pone.0259264)
Supplement: S1 Table — (DOCX) [file pone.0259264.s001.docx]

***S1 Table.*** *Prices for selected key agricultural goods in 2018 and 2020*

|  | **2018** | **2020** | **Difference (2020)-(2018)** | |
| --- | --- | --- | --- | --- |
|  | **Price per kg (Tk)** | **Price per kg (Tk)** |  |  |
| *Crop* | **Mean** | **Mean** |  | **(%)** |
| *Aman rice* | 20.8 | 24.2 | 3.4 | 16.35 |
| *Boro rice* | 21.4 | 23.9 | 2.5 | 11.68 |
| *Jute* | 34.7 | 56.8 | 22.1 | 63.69 |
| *Bringal* | 17.7 | 23.6 | 5.9 | 33.33 |
| *Potato* | 11.2 | 13.7 | 2.5 | 22.32 |
| *Sweetpotato* | 7.5 | 16.7 | 9.2 | 122.67 |

Note: USD 1 = TK 83.2 during the time of data collection in 2018/2020.
